# Adaptive Laboratory Evolution of *Eubacterium limosum* ATCC 8486 on Carbon Monoxide

**DOI:** 10.3389/fmicb.2020.00402

**Published:** 2020-03-11

**Authors:** Seulgi Kang, Yoseb Song, Sangrak Jin, Jongoh Shin, Jiyun Bae, Dong Rip Kim, Jung-Kul Lee, Sun Chang Kim, Suhyung Cho, Byung-Kwan Cho

**Affiliations:** ^1^Department of Biological Sciences, Korea Advanced Institute of Science and Technology, Daejeon, South Korea; ^2^KAIST Institute for the BioCentury, Korea Advanced Institute of Science and Technology, Daejeon, South Korea; ^3^Department of Mechanical Engineering, Hanyang University, Seoul, South Korea; ^4^Department of Chemical Engineering, Konkuk University, Seoul, South Korea; ^5^Intelligent Synthetic Biology Center, Daejeon, South Korea

**Keywords:** acetogens, carbon monoxide, adaptive laboratory evolution, CODH/ACS, *acsA*, *cooC*

## Abstract

Acetogens are naturally capable of metabolizing carbon monoxide (CO), a component of synthesis gas (syngas), for autotrophic growth in order to produce biomass and metabolites such as acetyl-CoA via the Wood–Ljungdahl pathway. However, the autotrophic growth of acetogens is often inhibited by the presence of high CO concentrations because of CO toxicity, thus limiting their biosynthetic potential for industrial applications. Herein, we implemented adaptive laboratory evolution (ALE) for growth improvement of *Eubacterium limosum* ATCC 8486 under high CO conditions. The strain evolved under syngas conditions with 44% CO over 150 generations, resulting in a significant increased optical density (600 nm) and growth rate by 2.14 and 1.44 folds, respectively. In addition, the evolved populations were capable of proliferating under CO concentrations as high as 80%. These results suggest that cell growth is enhanced as beneficial mutations are selected and accumulated, and the metabolism is altered to facilitate the enhanced phenotype. To identify the causal mutations related to growth improvement under high CO concentrations, we performed whole genome resequencing of each population at 50-generation intervals. Interestingly, we found key mutations in CO dehydrogenase/acetyl-CoA synthase (CODH/ACS) complex coding genes, *acsA* and *cooC*. To characterize the mutational effects on growth under CO, we isolated single clones and confirmed that the growth rate and CO tolerance level of the single clone were comparable to those of the evolved populations and wild type strain under CO conditions. Furthermore, the evolved strain produced 1.34 folds target metabolite acetoin when compared to the parental strain while introducing the biosynthetic pathway coding genes to the strains. Consequently, this study demonstrates that the mutations in the CODH/ACS complex affect autotrophic growth enhancement in the presence of CO as well as the CO tolerance of *E. limosum* ATCC 8486.

## Introduction

Carbon monoxide (CO), generated due to incomplete combustion of organic materials, is a toxic gas that hampers the growth of various organisms. Presently, CO is emitted in large quantities in the form of synthesis gas (syngas) comprising CO, carbon dioxide (CO_2_), and hydrogen (H_2_). The syngas is produced as a byproduct of fossil fuel combustion for industrial development, specifically by gasification of coal, biomass, and natural gas. The syngas composition depends on the gasifier type and resource, which increases the CO amount up to 67% of the total volume (Subramani and Gangwal, 2008; Munasinghe and Khanal, 2010). Being derived from fossil fuel, syngas needs to be purified in order to prevent air pollution and the greenhouse gas effect, which is conventionally managed via thermochemical processes that convert syngas into liquid hydrocarbons. Unfortunately, the conventional method requires greater operation cost and high temperature and pressure conditions, thus requiring a more efficient method to convert syngas into other chemicals (Bredwell et al., 1999; Munasinghe and Khanal, 2010). As an alternative method, gas fermentation using microorganisms has been suggested to produce industrial commodities with lower operation cost and higher catalyst specificity compared to the thermochemical processes. In addition, the biological process is capable of producing various organic compounds using syngas as feedstock, such as acetate, butyrate, ethanol, butanol, 2,3-butanediol, and other compounds via genetic manipulation (Köpke et al., 2010, 2011; Abubackar et al., 2015; Park et al., 2017). Among the promising biocatalysts for syngas fermentation, with an ability to convert CO into biomass and various biochemicals, acetogenic bacteria (acetogens) have received immense attention and are considered as a novel platform to replace the conventional processes (Henstra et al., 2007; Bengelsdorf et al., 2013; Latif et al., 2014).

Acetogens are anaerobic bacteria that utilize CO and CO_2_ as a carbon building block and, initially, synthesize acetyl-CoA as an important metabolic intermediate, by using the Wood–Ljungdahl pathway (WLP) (Drake et al., 2008). The linear WLP comprises two branches, methyl and carbonyl branches, which convert CO into CO_2_ and then to acetyl-CoA (Drake et al., 2006). The methyl branch reduces CO_2_ converted from CO into formate, catalyzed by formate dehydrogenase (*fdh*) (Ragsdale, 1997). Following the initial reaction, formyl-tetrahydrofolate (THF) is formed using formate and THF, which requires the hydrolysis of ATP (Schuchmann and Müller, 2014). Subsequently, the formyl-THF is converted by methenyl-THF cyclohydrolase into methenyl-THF, and further into methylene-THF via methylene-THF dehydrogenase. Eventually, the methyl branch reduces methylene-THF to methyl-THF by methylene-THF reductase (Ragsdale and Pierce, 2008). For the carbonyl branch, the methyl group of methyl-THF is transferred by methyltransferase to corrinoid Fe-S protein, and then to CO dehydrogenase/acetyl-CoA synthase (CODH/ACS), which carries CO generated by using CODH/ACS from CO_2_ (Ljungdahl, 1986). The condensation of methyl group and CO from the methyl and carbonyl branches, respectively, generates acetyl-CoA, which then converts into acetate by generating ATP (Ragsdale and Pierce, 2008). Of all the enzymes associated with the WLP, CODH/ACS plays a pivotal role in autotrophic growth of acetogens by reversibly interconverting CO/CO_2_ and synthesizing acetyl-CoA (Doukov et al., 2002).

In acetogens, the CODH/ACS complex is formed by the assembly of ACS and CODH, as (αβ)_2_ complex in the presence of [3Fe-4S] cluster (C-cluster), [4Fe-4S] cluster (A-cluster), and metal clusters as the active sites (Darnault et al., 2003; Ragsdale, 2008; Appel et al., 2013; Can et al., 2014). The C-cluster encoded by *acsA* is the active site of CODH subunit for reversible oxidation of CO to CO_2_ (Doukov et al., 2002). The A-cluster encoded *acsB* is the active site of ACS subunit, which generates acetyl-CoA from CO, CoA, and methyl group that is transferred from the corrinoid protein (Seravalli et al., 1997; Darnault et al., 2003; Drennan et al., 2004). For CO fixation of *Moorella thermoacetica*, for example, CO catalytic reaction is indicated as “ping-pong” reaction involving two steps, ping and pong step (Diekert and Thauer, 1978). In the ping step, CO binds to the metal center of the C-cluster, which is nickel, and thus reduces the C-cluster; thereafter, in the pong step, the electrons from the C-cluster are transferred to the external electron acceptors, such as ferredoxin, via the B- and D-clusters, and CO_2_ is generated by CO oxidation (Can et al., 2014). To activate this complex, specific accessory proteins, such as *cooC*, *cooJ*, or *cooT*, are required, which are responsible for binding the metal and forming metal binding site for the complex interface (Bender et al., 2011; Alfano et al., 2019). In addition, the proteins support maturation of CODH by assembling C-cluster in the CODH/ACS complex and transfer electrons obtained from CO oxidation to the electron carriers (Loke and Lindahl, 2003).

Using the WLP and the associated enzymes, acetogens utilize CO as a carbon substrate for producing biomass building blocks; however, they are inhibited by the high concentration of CO (Daniel et al., 1990). CO competitively binds to the active site of metalloenzyme, such as hydrogenase, and depletes the transition metal that leads to insufficient ligation of the original substrate, and the absence of metals causes low growth rate and eventually leads to mortality of the organism (Bertsch and Müller, 2015). For example, in *Acetobacterium woodii*, one of the well-known model acetogens, the growth rates under autotrophic growth conditions decreased with increasing CO concentrations, which also affected the heterotrophic growth conditions (Bertsch and Müller, 2015). CO inhibited hydrogen-dependent CO_2_ reductase of *A. woodii*, which is responsible for CO_2_ reduction and hydrogen storage (Bertsch and Müller, 2015). Although acetogens utilize CO as the carbon source, the inhibitory effect of high CO concentration on the growth and lethality of the organisms need to be enhanced for efficient CO fixation.

In the present study, we applied adaptive laboratory evolution (ALE) method to enhance CO tolerance and growth fitness of *Eubacterium limosum* ATCC 8486 under CO presence, by serially transferring the strain on syngas containing 44% CO for 150 generations. ALE is widely utilized, thus allowing self-optimization of the organism to acquire the desired phenotype (Elena and Lenski, 2003; Dragosits and Mattanovich, 2013; Choe et al., 2019). Genome sequencing of the evolved strains at 50-generation intervals revealed several causal mutations, which were identified in the genes encoding CODH/ACS. Subsequently, via the growth profiling of single isolated clone under syngas growth conditions, we validated that the key mutation altered the tolerance and the growth of the strain. The results provide insights on CO fixation for strain designing.

## Materials and Methods

### Bacterial Strains and Culture Conditions

*Eubacterium limosum* ATCC 8486 was obtained from the Leibniz Institute DSMZ-German Collection of Microorganisms and Cell Cultures (DSMZ, Braunschweig, Germany). The strain was grown strictly under anaerobic conditions at 37°C in 100 mL of modified DSMZ 135 medium (pH 7.0), which comprised 1 g/L ammonium chloride, 2 g/L yeast extract, 10 g/L sodium bicarbonate, 0.1 g/L magnesium sulfate heptahydrate, 0.3 g/L cysteine-HCl, 10 mL vitamin solution (4 mg/L biotin, 4 mg/L folic acid, 20 mg/L pyridoxine-HCl, 10 mg/L thiamine-HCl, 10 mg/L riboflavin, 10 mg/L nicotinic acid, 10 mg/L pantothenate, 0.2 mg/L vitamin B12, 10 mg/L *p*-aminobenzoic acid, and 10 mg/L lipoic acid), 5.36 mM K_2_HPO_4_, 4.64 mM KH_2_PO_4_, 4 μM resazurin, and 20 mL trace element solution (1.0 g/L nitrilotriacetic acid, 3.0 g/L MgSO_4_.7H_2_O, 0.5 g/L MnSO_4_.H_2_O, 1.0 g/L NaCl, 0.1 g/L FeSO_4_.7H_2_O, 180 mg/L CoSO_4_.7H_2_O, 0.1 g/L CaCl_2_.2H_2_O, 180 mg/L ZnSO_4_.7H_2_O, 10 mg/L CuSO_4_.5H_2_O, 20 mg/L KAI(SO_4_)_2_.12H_2_O, 10 mg/L H_3_BO_3_, 10 mg/L Na_2_MO_4_.2H_2_O, 30 mg/L NiCl_2_.6H_2_O, 0.3 mg/L Na_2_SeO_3_.5 H_2_O, 0.4 mg/L Na_2_WO_4_.2H_2_O), at a pressure of 200 kPa and 50 mL of headspace filled with 0%, 20%, 40%, 60%, 80%, and 100% CO that is balanced using 100%, 80%, 60%, 40%, 20%, and 0% N_2_, respectively, for autotrophic growth conditions. To enhance the autotrophic growth rate during adaptation, 40 mM NaCl was supplemented to the media, which couples with sodium dependent ATP synthase (Jeong et al., 2015; Song et al., 2017).

### Adaptive Laboratory Evolution

Adaptive laboratory evolution experiment was conducted with a syngas (44% CO, 22% CO_2_, 2% H_2_, and 32% N_2_) described in Section “Bacterial Strains and Culture Conditions.” Before the ALE, the strain underwent preadaptation step by passing thrice through the syngas condition at mid-exponential phase. During the ALE experiment with four independent populations, the culture was transferred to a fresh medium at mid-exponential phase.

### Whole Genome Resequencing

To construct whole genome resequencing libraries, the genomic DNA samples were extracted from the evolved populations and isolated single clone. Cell stocks were cultured in modified DSMZ 135 medium (pH 7.0) supplemented with glucose (5 g/L) and were incubated for 12 h at 37°C. The harvested cells were resuspended with 500 μL of lysis buffer containing Tris–HCl (pH 7.5), 5 M NaCl, 1 M MgCl_2_, and 20% triton × 100. Thereafter, the cells were frozen using liquid nitrogen and ground using a mortar and pestle. The powder was resuspended with 600 μL of nuclei lysis solution (Promega, Madison, WI, United States), incubated at 80°C, and then cooled at 4°C. RNAs were removed from the cell lysate using RNase A solution. Proteins in the solution were precipitated using protein precipitation buffer (Promega). Following the protein precipitation, the samples were cooled at 4°C for 10 min, and were then centrifuged at 16,000 *g* for 10 min. The supernatant was transferred to a new tube, and 1 × volume of isopropanol was added. After centrifugation at 16,000 *g* for 5 min, the DNA pellet was obtained, which was then washed with 80% ethanol twice. The obtained DNA quality was determined by the A260/A280 ratio (>1.9) and inspected by gel electrophoresis, and the concentration was quantified via a Qubit 2.0 Fluorometer (Invitrogen, Carlsbad, CA, United States) with a Qubit dsDNA HS Assay kit (Invitrogen). The sequencing libraries were constructed using a TruSeq Nano DNA library prep kit (Illumina, La Jolla, CA, United States). The constructed libraries of evolved populations were sequenced with Illumina HiSeq2500 (rapid-run mode as 50 cycle-ended reaction) and the constructed libraries of isolated clone were sequenced with Illumina MiSeq (a 150 cycle-ended reaction).

### Mutation Screening

All process for mutation screening were performed using a CLC genomics Workbench v6.5.1 (CLC bio, Aarhus, Denmark). Adapter sequences of the sequencing reads were removed by using a trimming tool with the default parameters (quality limit and ambiguous nucleotides residues 2). The resulting reads were mapped to the *E. limosum* ATCC 8486 reference genome (NCBI accession NZ_CP019962.1) with mapping parameters (mismatch cost: 2, indel cost: 3, deletion cost: 3, length fraction: 0.9, and similarity fraction: 0.9). Variants were detected from the mapped reads using a Quality-based variant detection tool with the parameters (neighborhood radius: 5, maximum gap and mismatch count: 5, minimum neighborhood quality: 30, minimum central quality: 30, minimum coverage: 10, minimum variant frequency: 10%, maximum expected alleles: 4, non-specific matches: ignore and genetic code: bacterial and plant plastid).

### Isolation of Single Clones From the Evolved Populations

The single clones were isolated from the evolved populations by streaking the culture onto RCM agar medium. To confirm the sequence of each mutation site, the genomic regions were amplified by PCR using primer pairs and sequenced by Sanger sequencing (Supplementary Table S1). The selected single clones with the mutations were cultured in DSMZ 135 medium supplemented with CO in the headspace to measure the growth rate and metabolite production.

### Plasmid Construction for Acetoin Biosynthesis

All primers used to construct plasmid in this study are listed in Supplementary Table S2. Initially, the pJIR750ai plasmid was used as a shuttle vector, and the chemically competent *Escherichia coli* DH5α (Enzynomics, Inc., South Korea) was used for cloning the plasmid. Acetolactate synthase (*alsS*) and acetolactate decarboxylase (*alsD*) were obtained via gene synthesis from *Bacillus subtilis* and *Aeromonas hydrophila*, respectively (Oliver et al., 2013). The synthesized *alsS* and *alsD* were amplified using primer sets alsS_F-alsS_R and alsD_F-alsD_R, respectively. The pJIR750ai reduced by *Pvu*I (named as pJIR750_*Pvu*I_cut) was digested by *Bam*HI and *Sal*I, and was then assembled with the amplified *alsD* by using In-Fusion HD cloning Kit (TaKaRa, Japan). Subsequently, the assembled plasmid (pJIR750_alsD) was linearized by *Sac*I and *Bam*HI, and was then assembled with the amplified *alsS* by In-Fusion cloning Kit, generating the pJIR750_alsS_alsD plasmid. To control the gene expressions, promoters of ELIM_c2885 (pyruvate:ferredoxin oxidoreductase) and ELIM_c1121 ([Fe] hydrogenase) were selected as the two genes were constitutively expressed with high expression levels in *E. limosum* (Song et al., 2018). The native promoters were amplified from genomic DNA of *E. limosum* and inserted in the pJIR750_alsS_alsD plasmid, resulting in the construction of pJIR750_alsS_U_1121_P1121_P2885_U1121_alsD plasmid.

### Transformation

To prepare the electrocompetent strains, a previously modified protocol was used (Shin et al., 2019). The cells were cultured in 100 mL of DSM 135 medium supplemented with 5 g/L glucose. At the early exponential phase (OD_600_ 0.3 ∼ 0.5), the cells were harvested by centrifuging at 10,000 rpm for 10 min at 4°C. The harvested cells were washed with 50 mL of 270 mM sucrose buffer (pH 6) and resuspended to achieve a final concentration of 10^11^ cells/mL. About 1.5 ∼ 2 μg plasmid was added to the electrocompetent cells and then the solution was transferred to a 0.1-cm-gap Gene Pulser cuvette (Bio-Rad, Hercules, CA, United States). Thereafter, the cells were pulsed at 2.0 kV and immediately resuspended with 0.9 mL of reinforced clostridial medium (RCM). The cells were recovered on ice for 5 min, and incubated at 37°C for 16 h. The recovered cells were plated on an RCM plate (1.5% agar) containing 15 μg/mL thiamphenicol. A single colony was selected and cultured in DSM 135 medium supplemented with 5 g/L glucose.

### Metabolite Measurement

Primary metabolites were measured via high performance liquid chromatography (Waters, Milford, MA, United States) equipped with refractive index detector and MetaCarb 87 H 300 × 7.8 mm column (Agilent, Santa Clara, CA, United States). The mobile phase used was 0.007 N sulfuric acid solution with 0.6 mL/min flow rate. The oven temperature was set at 37°C for acetate, lactate and butyrate and 50°C for acetoin.

### Gas Measurement

CO and CO_2_ concentrations were measured via gas chromatography (Shimadzu, Japan) equipped with thermal conductivity detector and ShinCarbon ST Micropacked column (1 mm × 2 m, 1/16′′, 100/120 mesh; Restek, Bellefonte, PA, United States). Helium was used as the carrier gas at a flow rate of 30 mL/min. The initial oven temperature was 30°C for 1 min, programmed with a ratio 5°C/min until it reached 100°C. The temperature for injector and detector was 100°C.

## Results

### Growth of *E. limosum* ATCC 8486 Under CO Culture Conditions

**FIGURE 1:**
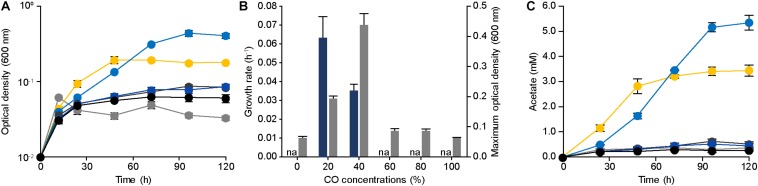
Physiology of *Eubacterium limosum* ATCC 8486 with different amounts of CO as sole substrate and source of energy. **(A)** The growth profiles of the parental strain under 0% (gray circle), 20% (yellow circle), 40% (light blue circle), 60% (dark gray circle), 80% (blue circle), and 100% (black circle) CO conditions that are balanced by N_2_ were measured using optical density at 600 nm. **(B)** The growth rates (navy) and the maximum optical density (600 nm) (gray) of the parental strain under 0%, 20%, 40%, 60%, 80%, and 100% CO that is balanced by N_2_. “na” is not available. **(C)** Acetate productions by the parental strain under 0% (gray circle), 20% (yellow circle), 40% (light blue circle), 60% (dark gray circle), 80% (blue circle), and 100% (black circle) CO conditions that are balanced by N_2_ were measured using HPLC, respectively. The cells were anaerobically cultivated at 37°C in 150 mL anaerobic bottle containing 100 mL of the modified DSMZ 135 medium with 50 mL of headspace purged at a pressure of 200 kPa. Error bars indicate standard deviation of biological triplicates.

To confirm the CO tolerance of *E. limosum* ATCC 8486, the cell growth was determined by culturing the strain in 100 mL of the modified DSMZ 135 medium with 0%, 20%, 40%, 60%, 80%, and 100% CO concentrations (Figure 1A). In the absence of CO, *E. limosum* proliferated to a maximum optical density at 600 nm of 0.062 ± 0.003, which likely due to the presence of sodium bicarbonate and yeast extract in the medium served as the carbon source for the *E. limosum* (Figure 1B). Under 20% CO growth condition, the cell reached a growth rate of 0.063 ± 0.011 h^–1^; whereas, a growth rate of 40% CO growth condition was 0.035 ± 0.002 h^–1^, which increased by 1.80 folds. In contrast, maximum optical densities (600 nm) of 20% CO and 40% CO culture conditions were 0.193 and 0.438, respectively, which decreased by 2.27 folds (Figure 1B). The cell cultivated under CO concentrations exceeding 60% demonstrated insignificant proliferation under the conditions, indicating that cell growth was inhibited with increasing CO concentrations in the growth medium. We performed an additional experiment to confirm the effect of CO on the cell growth. Initially, the cells were cultured in the DSMZ 135 medium containing 5 g/L glucose, then incubated until reaching the mid-exponential phase, optical density (600 nm) of 1.320. When the cell reached the mid-exponential phase, 0% (100% N_2_, as control), 20%, 40%, 60%, 80%, or 100% CO was purged to each sample with the same pressure, then measured the optical density (600 nm) of the cells (Supplementary Figure S1). Two hours after the CO injection, the optical densities (600 nm) were measured, which decreased compared to the control at all CO concentrations (Supplementary Figure S1).

To further understand the phenotypical effect of CO, metabolites produced by *E. limosum* were investigated (Figure 1C). Among the identified metabolites, acetate was the dominant metabolic product, which is known as a key end product for acetogens under autotrophic growth conditions. In general, acetate production is well correlated with the cell growth, because acetate synthesis generates ATP that is required for cellular function of acetogens. Under 20% and 40% CO conditions, the acetate production was increased compared to the other CO conditions; moreover, the acetate production patterns revealed growth-dependent profiles. Under high CO conditions, acetate production decreased as the concentrations of CO increased, and insignificant changes were determined (*P*-value > 0.05) compared to the control experiment. Based on these results, the acetate productions were correlated with the growth pattern, which was influenced by the amount of CO concentration in the culture medium, suggesting that the increase of CO tolerance potentially enhances *E. limosum* acetate production.

### Adaptive Laboratory Evolution of *E. limosum* ATCC 8486 Under CO Culture Conditions

**FIGURE 2:**
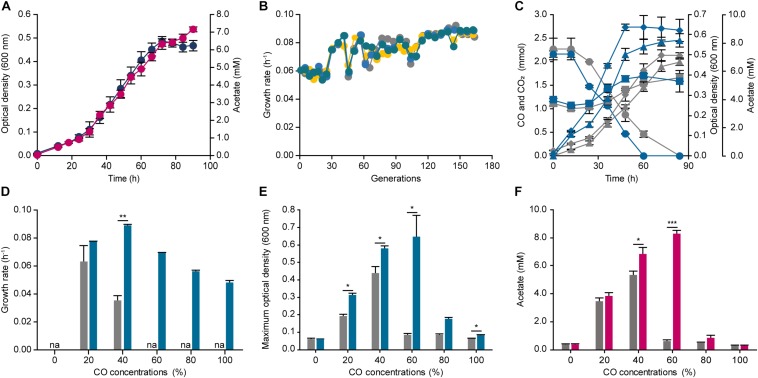
Adaptive laboratory evolution of *Eubacterium limosum* ATCC 8486 under 44% CO syngas condition. **(A)** Optical density (600 nm) (navy circle) and acetate production (pink circle) of *E. limosum* under 44% CO syngas condition were measured over 90 h. **(B)** Changes of four independent population growth rates, ALE1 (gray circle), ALE2 (yellow circle), ALE3 (blue circle), and ALE4 (bluish green circle), during ALE under 44% CO syngas condition. Each circle indicates passage points during ALE. **(C)** The profile of CO consumptions (circle), CO_2_ (square), acetate generation (triangle), and optical densities (600 nm) (diamond) of the evolved populations (bluish green lines) compared to the parental strain (gray lines) under 44% CO syngas condition. **(D)** The growth rates of ECO (bluish green bar) compared to the parental strain (gray bar) under 0%, 20%, 40%, 60%, 80%, and 100% CO that is balanced by N_2_. “na” is not available. **(E)** The maximum optical densities (600 nm) of the parental strain (gray bar) and ECO (bluish green bar) under 0%, 20%, 40%, 60%, 80%, and 100% CO, which balanced by N_2_. **(F)** Acetate production of ECO (pink bar) compared to the parental strain (gray bar) under 0%, 20%, 40%, 60%, 80%, and 100% CO that is balanced by N_2_. The cells were anaerobically cultivated at 37°C in 150 mL anaerobic bottle containing 100 mL of the modified DSMZ 135 medium with 50 mL of headspace purged at a pressure of 200 kPa. Error bars indicate standard deviation of biological triplicates. Asterisks indicates as following: *: *P*-value ≤ 0.05, **: *P*-value ≤ 0.01, ***: *P*-value ≤ 0.001.

To improve the CO tolerance of *E. limosum*, we applied the robust ALE method to the organism, which is used as a tool to engineer organism for overcoming target stress conditions. For designing the ALE experiment, establishing an appropriate stress condition is an essential factor for an organism to reach the desired phenotype. In this study, syngas was selected for the ALE experiment. Syngas is generated from gasification, and the composition of the gas is determined by a source of gasifier type or biomass, which typically comprises CO and H_2_ with 14% ∼ 67% and 5% ∼ 32%, respectively (Munasinghe and Khanal, 2010). To decide the precise composition of syngas for ALE, previous studies on syngas fermentations of acetogens were investigated, and a syngas composition of 44% CO, 22% CO_2_, 2% H_2_, and balanced N_2_ was selected, which was widely observed in the syngas generated by industries (Köpke et al., 2011). Using the syngas composition, *E. limosum* was cultivated in the same basal media, which was utilized for determining the CO tolerance, to determine the transfer point for ALE (Figure 2A). The growth rate under the condition was 0.070 ± 0.002 h^–1^ with a maximum optical density (600 nm) of 0.486 ± 0.021, which slightly differs compared to the growth rate (0.058 ± 0.000 h^–1^) obtained from the 40% CO condition, because a presence of H_2_ with CO in the environment enhances the autotrophic growth of acetogens (Bertsch and Müller, 2015; Valgepea et al., 2018). According to the growth profile, the mid-exponential phase is between 42 and 54 h after the initial inoculation; thus, we selected 48 h for the transfer point for ALE. For ALE, four separate populations of *E. limosum* were adapted to confirm the reproducibility, labeling as ALE1, 2, 3, and 4. Initially, at the 40^th^ generation, the growth rates of all populations were increased to 0.085 h^–1^, which then maintained the enhanced growth rate up to 120^th^ generation (Figure 2B). After 120 generations of adaptation, slight variations in the growth rates were observed in the entire population, with growth rate around 0.086 h^–1^; however, no growth rate changes were observed after 150 generations (Figure 2B); thus, the ALE was stopped at 150^th^ generation.

To further investigate the changes in cell growth at the population level, we selected the evolved population (ECO), ALE4, which demonstrated the highest growth rate with 0.089 h^–1^ at the 150^th^ generation. Moreover, the other growth rates were comparable to that of the ECO, which were 0.086, 0.087, and 0.088 h^–1^ for the ALE1, ALE2, and ALE3, respectively. Growth and CO consumption profiles of the ECO and the parental strain were compared under the syngas growth conditions (Figure 2C). The two strains completely consumed CO presented in the headspace by 84 and 60 h, respectively, resulting in CO consumption rates of 0.043 ± 0.019 and 0.058 ± 0.003 mmol h^–1^, respectively, which increased the carbon consumption rate of the adapted strain by 1.35 folds (Figure 2C). In addition, the stationary phases of the parental strain and the ECO were attained at 72 and 48 h, respectively, and the maximum optical densities (600 nm) were 0.498 ± 0.028 and 0.639 ± 0.016, respectively, which increased by 1.28 folds (Figure 2C).

Following the investigation of its phenotypical changes under the syngas condition, CO tolerance of the ECO was measured by cultivating the strain under different CO concentrations ranging from 0% to 100% CO (Figure 2D). Under 20% and 40% CO conditions, the growth rates were 0.076 ± 0.001 and 0.089 ± 0.001 h^–1^, respectively. Compared to the parental strain, the growth rates of the ECO under 20% and 40% CO conditions were enhanced by 1.21 and 1.65 folds higher. In addition, the growth rates of the inhibitory CO conditions were 0.069 ± 0.001, 0.056 ± 0.001, and 0.048 ± 0.001 h^–1^ under 60%, 80%, and 100% CO conditions, respectively (Figure 2D). In addition, the maximum optical density at 600 nm of the ECO were higher than that of the parental strain by 7.49 folds under 60% CO condition (Figure 2E). Acetate productions of the ECO were identified under the given conditions, resulting that 0.274 ± 0.060, 3.835 ± 0.215, 6.819 ± 0.457, 8.311 ± 0.254, 0.866 ± 0.217, and 0.264 ± 0.043 mM of acetate were produced under 0%, 20%, 40%, 60%, 80%, and 100% CO conditions (Figure 2F). In general, the acetate productions of the ECO were growth dependent, similar to those of the parental strain; however, they were increased compared to the production by the parental strain. Such results indicate that the adaptively evolved *E. limosum* enhanced CO consumption, growth, and tolerance under the autotrophic conditions, thus questioning about genetic mutations causing the phenotypic changes.

### Mutation Profiles by Whole Genome Resequencing

Adaptive laboratory evolution allows a target organism to adapt to the desired condition by modifying the genotype, rewiring and changing the metabolic pathways, and altering enzyme kinetics in the organism. Identification of mutations in the genome throughout the ALE is important to understand the fitness landscape of the organism under the growth condition. Although the ALE revealed that the fitness, tolerance, and acetate production were increased under the 44% CO condition, the causal genotypic changes and their collateral effects on the phenotype remain unclear. To address this, the four evolved populations at initial, 50^th^, 100^th^, and 150^th^ generation were proceeded for whole genome resequencing. We identified 39 mutations in all adapted strains, locating 33 and 6 mutations in the genic and intergenic regions, respectively (Supplementary Table S3).

Among these, five common mutations were identified across the populations, resulting in five key mutations with top 15% mutation frequency in the evolved populations. The five mutations were located in ELIM_c1038, ELIM_c1073, and ELIM_c1653 as single nucleotide variations (SNVs) and ELIM_c1031 and ELIM_c1654 as nucleotide insertion mutations (Table 1). For SNVs, E^48^K in ELIM_c1038, Y^136^X in ELIM_c1073, and A^97^E in ELIM_c1653 were identified, which are responsible for putative ATPase, DNA methylase (*dam*), and CODH catalytic subunit (*acsA*), respectively. For the insertional mutations, N^119^KfsX133 in ELIM_c1031 encoding integrase protein and A^72^AfsX92 in ELIM_c1654 encoding CODH accessory protein (*cooC2*) were observed. Of the five mutated genes, two mutations were located in the putative genes, and interestingly the other three mutations were located in the genes with certain functional roles, of which two mutations occurred in CODH/ACS complex coding genes, *acsA* and *cooC2*, that were reported to play a pivotal role for the active site and maturity of the complex (Morton et al., 1991; Kerby et al., 1997; Bender et al., 2011). The mutation in *acsA* was not located at the activate sites of the enzyme; however, substituting a small non-polar amino acid into a large polar amino acid potentially altered the protein structure that affects the enzymatic activity. The other mutation, which occurred in *cooC2*, introduced an early stop codon at 20^th^ amino acid downstream of the mutation site, which revealed synonymous substitution at the mutation site. The early termination often leads to a loss of function, suggesting that, despite the importance of *cooC* under the autotrophic growth condition, the functional role of the gene in the evolved strain may be ineffective. Collectively, the five mutations were the dominant variants, and three mutations were located in the genes with functional roles, of which *acsA* was hypothesized to be the driving mutation for the altered phenotype in adapted strain under autotrophic condition.

**TABLE 1:** Key mutations in the evolved populations.

Locus tag	Gene	Mutation (Type)	AA change	Description
ELIM_c1031	–	−356T (insertion)	Asn^119^LysfsX133	Integrase family protein
ELIM_c1038	–	G133A (SNV)	Glu^48^Lys	Putative ATPase, transposase-like protein
ELIM_c1073	*dam*	T408G (SNV)	Tyr^136^X	N6 adenine-specific DNA methylase D12 class
ELIM_c1653	*acsA*	C290A (SNV)	Ala^97^Glu	CODH catalytic subunit
ELIM_c1654	*cooC2*	−216A (insertion)	Ala^72^AlafsX92	CODH nickel insertion accessory protein

### Effect of the Mutation in *acsA* on CO Fixation

In order to validate the hypothesis, obtaining a strain from ECO with the mutation on *acsA* is essential. For obtaining a single clone with the *acsA* mutation from the evolved populations, 20 colonies were isolated, which then confirmed the presence of *acsA* mutation in 17 of 20 colonies. Of the obtained 17 colonies, a single clone with the *acsA* mutation and without the other key mutations was selected, labeling as ECO_acsA strain (Supplementary Figure S2). The obtained strain, ECO_acsA, underwent genome resequencing to identify the mutations embedded in the genome, resulting in six mutations with four in the genic regions and two in the intergenic regions (Supplementary Table S4). The four mutations were associated with ELIM_c0006, ELIM_c1653, ELIM_c2214, and ELIM_c2227. Of the four mutations, only ELIM_c1653, which encodes *acsA*, is associated with the autotrophic growth condition.

**FIGURE 3:**
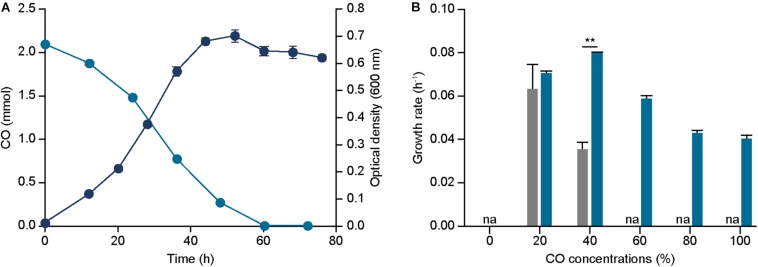
Effect of *acsA* mutation on growth and CO tolerance. **(A)** The profile of CO consumptions (bluish green circle), and optical densities (600 nm) (navy circle) of ECO_acsA under 44% CO syngas. **(B)** The growth rates of ECO_acsA (bluish green bar) and parental strain (gray bar) under 0%, 20%, 40%, 60%, 80%, and 100% CO conditions that are balanced by N_2_. The cells were anaerobically cultivated at 37°C in 150 mL anaerobic bottle containing 100 mL of the modified DSMZ 135 medium with 50 mL of headspace purged at a pressure of 200 kPa. “na” is not available. Error bars indicate standard deviation of three biological replicates. Asterisks indicates as following: **: *P*-value ≤ 0.01.

Initially, growth profile of the strain under syngas condition with 44% CO was measured and compared to the parental strain, resulting in a growth rate of 0.095 ± 0.000 h^–1^ and 0.050 ± 0.001 h^–1^ and a maximum optical density (600 nm) of 0.703 ± 0.023 and 0.498 ± 0.028 for ECO_acsA and parental strain, respectively (Figure 3A). These results indicate that ECO_acsA strain proliferates more rapidly by 1.90 folds with higher optical densities (600 nm) by 1.41 folds than the parental strain under CO condition.

To further understand the phenotypic changes, CO consumed and metabolites produced by the ECO_acsA strain were measured under the syngas condition with 44% CO. In accordance to the previous analysis on metabolites, acetate was the dominant end product produced by the ECO_acsA strain, with 6.889 mM. Increase in the acetate production by the strain indicates that the altered C-cluster of the active site of CODH subunit encoded by the mutated *acsA* enhanced CO utilization (Figure 3A). Consistent with acetate production, the CO consumption rates by ECO_acsA and parental strain were 0.059 ± 0.002 and 0.043 ± 0.019 mmol h^–1^, respectively, which differed by 1.37 folds (Figure 3A).

Although the growth rate under CO condition was enhanced, the increase in CO tolerance of ECO_acsA strain compared to the parental strain remains unclear. To measure the CO tolerance, the strain was cultured under different CO concentrations. The growth rates of ECO_acsA strain under 20% and 40% CO conditions were 0.070 ± 0.001 and 0.079 ± 0.001 h^–1^, and these decreased for the strains cultured under high CO conditions with 0.059 ± 0.001, 0.043 ± 0.002, and 0.040 ± 0.001 h^–1^ for 60%, 80%, and 100% CO, respectively (Figure 3B). Although the growth rates decreased with increasing CO concentrations, compared to the parental strain, the growth rates of the ECO_acsA strain were increased by 2.25 folds under 40% CO, and the maximum optical density (600 nm) under all CO concentrations (Supplementary Figure S3), representing CO tolerance of the strain was greatly enhanced. Based on these results, our hypothesis, in which the mutation on *acsA* affects phenotype of the *E. limosum* under CO growth conditions, was tested through growth profiling and metabolite measurement experiments. We conclude that, indeed, the speculation was correct with enhanced growth rate, CO consumption rate, acetate production, and CO tolerance under CO growth conditions, suggesting that mutation on *acsA* is essential to engineer the strain for CO utilization.

### Acetoin Production by ECO_acsA Strain

The ECO_acsA strain demonstrated the enhanced CO consumption and growth rates, thereby querying whether the evolved strain is a better platform to produce biochemical than the parental strain. To investigate the strain capacity to produce biochemicals, acetoin was selected as a target chemical, which is widely utilized in various industries from cosmetics, food flavoring, and pharmaceuticals (Werpy and Petersen, 2004; Bao et al., 2015). Interestingly, in *E. limosum*, the acetoin biosynthetic pathway coding genes are located, but the production was not detected under CO and other conditions, such as glucose and H_2_/CO_2_ conditions (Song et al., 2017, 2018). In the presence of the genes, *E. limosum* is capable of synthesizing acetoin; however, the insignificant transcriptional and translational expressions under the heterotrophic and autotrophic conditions prevent the production, according to a previous study (Song et al., 2018). Thus, activation of the biosynthetic pathway coding genes using novel bio-parts potentially produce acetoin in *E. limosum*, and then determine whether ECO_acsA is a superior platform to produce the biochemical compounds.

**FIGURE 4:**
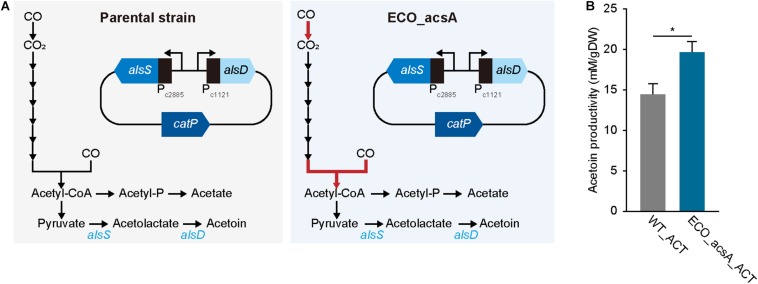
Acetoin production by *Eubacterium limosum*. **(A)** Metabolic pathway from CO to acetoin, in presence of the acetoin biosynthesis carrying plasmid. The constructed plasmid was transformed to ECO_acsA and the parental strain, which was named ECO_acsA_ACT and WT_ACT, respectively. The red arrow indicates CODH/ACS reaction encoded by *acsA*. **(B)** The production of acetoin by ECO_acsA_ACT (bluish green bar) and the WT_ACT (gray bar). The cells were anaerobically cultivated at 37°C in 150 mL anaerobic bottle containing 100 mL of the modified DSMZ 135 medium with 50 mL of headspace purged at a pressure of 200 kPa. Error bars indicate standard deviation of biological triplicates. Asterisks indicates as following: *: *P*-value ≤ 0.05.

To produce acetoin, a plasmid with *alsS*, which encodes α-acetolactate synthase that condenses two molecules of pyruvate to one acetolactate, and *alsD*, which encodes acetolactate decarboxylase that converts acetolactate to acetoin, was constructed (Figure 4A and see “Materials and methods” for more details). For activating the biosynthesis, bio-parts associated with highly expressed genes that are functionally related to CO metabolism were selected, locating promoters of ELIM_c2885 encoding pyruvate:ferredoxin oxidoreductase and ELIM_c1121 encoding hydrogenase subunit for *alsS* and *alsD*, respectively, with 5′ untranslated region that was obtained from ELIM_c1121 (Figure 4A; Song et al., 2018). Following the construction, the plasmid was transformed into the parental strain, which was then cultured for biological triplicates under the syngas condition with 44% CO, proliferating with cell density of 0.053 ± 0.002 gDW and producing acetoin of 14.6 ± 0.8 mM/gDW, along with 23.3 ± 1.5 mM/gDW acetate, 4.0 ± 0.0 mM/gDW butyrate, and 6.2 ± 1.7 mM/gDW lactate, which recovered 96.2% of carbons (Figure 4B). After confirming acetoin production, the same plasmid was introduced into ECO_acsA strain, and was then cultured under similar CO condition; thereafter, the acetoin production was measured, resulting in 19.6 ± 1.3 mM/gDW, which significantly increased by 1.34 folds (*P*-value ≤ 0.015). In addition, ECO_acsA strain with cell density of 0.034 ± 0.001 gDW produced 23.6 ± 1.1 mM/gDW acetate, 6.4 ± 0.2 mM/gDW butyrate, and 0.7 ± 0.5 mM/gDW lactate, which recovering 86.7% of carbons. Regarding CO consumption, the WT_ACT strain consumed with 429.8 ± 131.6 mM/gDW and ECO_acsA_ACT strain consumed 642.7 ± 317.8 mM/gDW, indicating higher CO consumption value by ECO_acsA_ACT strain compared to that by WT_ACT strain. Less consumption of CO leads to needing for the reduced ferredoxin, which is essential for *E. limosum* under autotrophic growth condition by generating a chemiosmosis gradient that synthesizes ATP. Depletion of reduced ferredoxin can be replenished by producing lactate from pyruvate via lactate dehydrogenase reaction, which oxidizes NADH and reduces ferredoxin. Based on the assumption, compared to ECO_acsA_ACT strain, the WT_ACT strain consumed less CO that caused less available reduced ferredoxin. To overcome the needs, the WT_ACT strain produced more lactate by catalyzing lactate dehydrogenase that produces reduced ferredoxin. Whereas, with the increased CO consumption capacity and more reduced ferredoxin available, ECO_acsA_ACT strain does not need to catalyze lactate dehydrogenase and alter the carbon flux to produce acetoin. Therefore, the authors hypothesize that the ECO_acsA strain produced significantly enhanced the amount of acetoin, using increased capacity with increased CO consumption and CO tolerance. Further studies on metabolic engineering are needed to verify the effects of the mutations on the change of the production by the acetogens.

## Discussion

Converting CO into biofuels and biochemicals provides several advantages such as low feedstock cost, utilization of harmful gas, and reduction of climate-changing substrate. Despite the advantages, CO has been a challenging feedstock due to lack of suitable microorganisms that tolerate and utilize CO as a substrate. Among the candidate organisms, acetogens have been suggested as a crucial platform to convert CO into various biochemicals using the WLP that oxidizes CO into CO_2_, and then into an important major metabolite, acetyl-CoA. All acetogens carry the unique pathway to synthesize acetyl-CoA under autotrophic growth conditions; however, the growth is inhibited under high CO concentrations, indicating that CO utilizing acetogens are intolerant toward CO. According to the previous studies, growth of *A. woodii*, a model acetogen that is phylogenetically related to *E. limosum*, is completely inhibited by the presence of CO with higher than 25% in the culture headspace (Bertsch and Müller, 2015). In this study, using ALE, the growth rate and CO tolerance of *E. limosum* were enhanced, which is highly related with the previous studies on *Thermoanaerobacter kivui* and *Butyribacterium methylotrophicum*. The studies reported that passaging the strains under CO conditions few times enhanced the utilization of CO as a feedstock, suggesting that wild type acetogens under CO conditions are not optimal and further enhancement is possible via ALE (Lynd et al., 1982; Weghoff and Müller, 2016). The enhanced growth rate of *E. limosum* was higher than that of other acetogens under CO conditions, including the growth rates of the adjusted acetogens (Table 2). Prior to this study, the highest growth rate under CO growth condition was that of *Clostridium carboxidivorans* (0.084 h^–1^), followed by *T. kivui* (0.068 h^–1^) and *C. ljungdahlii* (0.060 h^–1^), which are known as CO utilizing organisms (Phillips et al., 1994; Fernandez-Naveira et al., 2016; Weghoff and Müller, 2016). The growth rates of *E. limosum* under CO condition changed from 0.058 to 0.089 h^–1^ and increased by 1.53 folds through the ALE, and the growth rate under 100% CO condition was 0.048 h^–1^, thus indicating that *E. limosum* is one of the fastest growing acetogen strains under the autotrophic growth condition.

**TABLE 2:** Comparison of the growth rates of acetogens.

Strain	Gas condition	Growth rate	References
*Acetobacterium woodii*	5% CO/16% CO_2_/64% H_2_ (100 kPa)	0.028 h^–1^	Bertsch and Müller, 2015
	10% CO/16% CO_2_/64% H_2_ (100 kPa)	∼ 0.022 h^–1^	
	15% CO/16% CO_2_/64% H_2_ (100 kPa)	∼ 0.011 h^–1^	
	25% CO/15% CO_2_ (100 kPa)	No growth	
*Butyribacterium methylotrophicum*	100% CO (100 kPa)	0.050 h^–1^	Lynd et al., 1982
*Clostridium autoethanogenum*	45% CO/20% CO_2_/2% H_2_ (200 kPa)*	0.057 ± 0.04 h^–1^	Marcellin et al., 2016
	100% CO (200 kPa)	0.019 h^–1^	Liew et al., 2016
*Clostridium carboxidivorans*	100% CO (120 kPa)	0.084 ± 0.004 h^–1^	Fernandez-Naveira et al., 2016
*Clostridium ljungdahlii*	80% CO/20% CO_2_ (200 kPa)	0.060 h^–1^	Phillips et al., 1994
*Eubacterium limosum*	44% CO/22% CO_2_/2% H_2_ (200 kPa)*	0.095 ± 0.000 h^–1^	This study
*Moorella thermoacetica*	30% CO/30% CO_2_ (240 kPa)	0.069 h^–1^	Daniel et al., 1990
*Thermoanaerobacter kivui*	CO 20% (200 kPa) Makeup gas (80% N_2_/20% CO_2_)	0.037 h^–1^	Weghoff and Müller, 2016
	CO 50% (200 kPa) Makeup gas (80% N_2_/20% CO_2_)	0.045 h^–1^	
	CO 70% (200 kPa) Makeup gas (80% N_2_/20% CO_2_)	0.068 h^–1^	
	CO 90% (200 kPa) Makeup gas (80% N_2_/20% CO_2_)	0.020 h^–1^	
	CO 100% (200 kPa)	0.021 h^–1^	

*Industrial syngas composition.

Understanding the genomes of phenotypically altered organisms reveals a relationship between the genotype and phenotype. Genome resequencing of the evolved *E. limosum* identified five key mutations sites in the genome. Specifically, mutations on *acsA* and *cooC2* were in accordance with the previous understanding on CO oxidization mechanism that CODH/ACS complex plays a vital role under the CO fixing condition. However, the mutation on *cooC2*, which is crucial for CO oxidation by activating CODH/ACS protein complex by binding to the essential metals, contradicts our hypothesis by introducing an early stop codon at 20 sequences downstream of the mutation site, thus reducing the protein comprising 261 amino acids to 92 amino acids. The insertion of early stop codon prevents translation of the *cooC2* active site that is located at Cys116 and Cys118, which are the conserved sites for metal binding, leading to loss of function. In the previous study (Merrouch et al., 2018), increase in the *cooC* expression elevated CODH activity only in media without nickel supplementation; however, in the present study, nickel was supplemented in the media, making *cooC2* unessential under the condition that led to an introduction of a stop codon for the loss of function; whereas, a mutation on *acsA*, which is responsible for C-cluster of CODH/ACS complex, potentially altered the protein structure.

In the evolved strain, ECO, metabolite production pattern changed compared to the wild type *E. limosum*. In the wild type, acetate was majorly produced, which was similar for the ECO. Interestingly, the ECO, with higher growth rate compared to wild type, produced butyrate under CO condition, with acetate as the major metabolite. Despite the similarity at the genomic level between the *E. limosum* strains (ATCC 8486 and KIST612), butyrate production was not observed for wild type *E. limosum* ATCC 8486 under the CO condition, thus contradicting the previous report on *E. limosum* KIST612 (Chang et al., 1998). *E. limosum* KIST612 produced acetate and butyrate under CO condition (Jeong et al., 2015). For butyrate production, three additional reduction powers are required that recycle the excessive reducing equivalents, with potential ATP production (Kerby et al., 1997). CO oxidation generates reducing equivalent that needs to be oxidized, often utilized for reducing WLP enzymes to convert carbons and pumping ions across the membrane to create a chemiosmotic gradient for ATP synthesis. Based on the phenotypic results, the ECO altered the metabolite pathway to produce butyrate for oxidizing excessive reduction power and generating ATP, which potentially oxidizes CO faster with more available oxidized electron carriers that needs to be further validated (Supplementary Figure S4). Overall, we developed *E. limosum* strain that tolerates and efficiently utilizes CO as feedstock via ALE, then identified a key mutation on *acsA* encoding a subunit of CODH/ACS complex that caused phenotypic traits, and thereafter validated the hypothesis through phenotypic assays. Eventually, we utilized the ECO_acsA strain to construct an engineered strain to produce biochemical using CO as carbon source. The results will serve as an important resource for optimizing CO fermentation and strain designing for better biochemical production.

## Data Availability Statement

The whole genome resequencing data analyzed by this study are available in the EMBL European Nucleotide Archive (ENA) with Primary accession number PRJEB34640.

## Author Contributions

B-KC conceived and supervised the study. SK, SJ, JS, and B-KC designed the experiments. SK, SJ, and JB performed the experiments. SK, YS, SJ, SC, J-KL, DK, SCK, and B-KC analyzed the data. SK, YS, SC, and B-KC wrote the manuscript. All authors read and approved the final manuscript.

## Conflict of Interest

The authors declare that the research was conducted in the absence of any commercial or financial relationships that could be construed as a potential conflict of interest.
