# Supplementary material for: Adaptive Laboratory Evolution of Eubacterium limosum ATCC 8486 on Carbon Monoxide
Source: Front Microbiol. 2020 Mar 11;11:402. doi: 10.3389/fmicb.2020.00402 (PMC7079680; doi:10.3389/fmicb.2020.00402)
Supplement: Supplementary file 1 [file Data_Sheet_1.DOCX]

Supplementary Material

Adaptive laboratory evolution of *Eubacterium limosum* ATCC 8486 on carbon monoxide

Seulgi Kang^1,†^, Yoseb Song^1,†^, Sangrak Jin^1^, Jongoh Shin^1^, Jiyun Bae^1^, Dong Rip Kim^2^, Jung-Kul Lee^3^, Sun Chang Kim^1,4^, Suhyung Cho^1^, and Byung-Kwan Cho^1,4*^

*** Correspondence:** Byung-Kwan Cho, bcho@kaist.ac.kr

^†^ These authors contributed equally to this work.


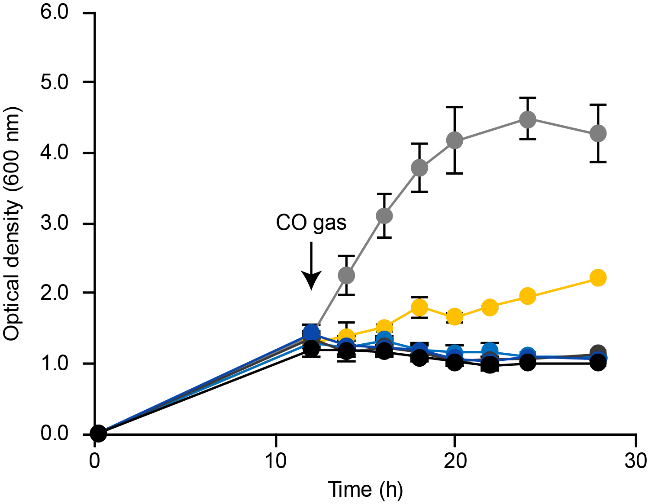


**Supplementary Figure S1**. The growth of the parental strain under CO condition with glucose. The parental strain was cultured under glucose (5 g/L) condition until mix-exponential phase (OD_600 nm_ 1.320). At the point of mid-exponential growth, 0% (gray circle), 20% (yellow circle), 40% (light blue circle), 60% (dark gray circle), 80% (blue circle), and 100% (black circle) CO (balanced with N_2_ gas) were injected in the headspace of culture bottles. Each circle indicates sampling point for the optical density (600 nm) measurement.


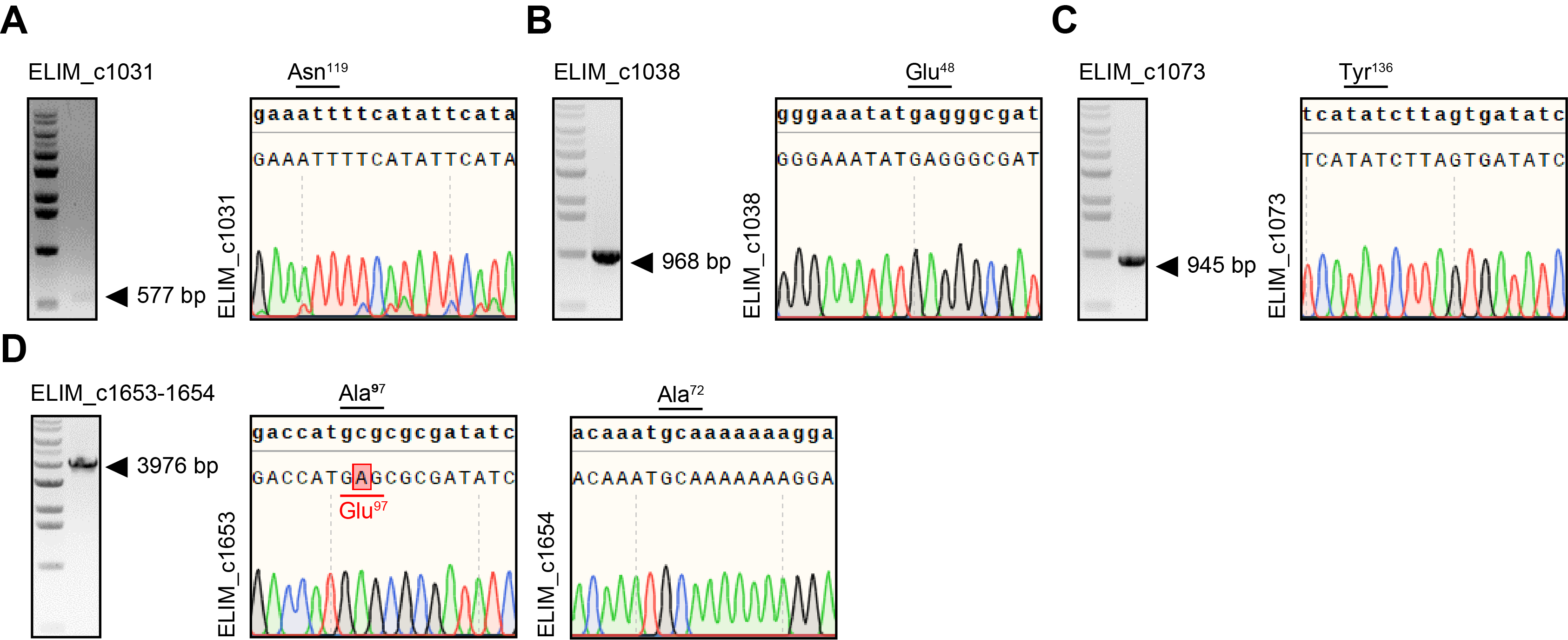


**Supplementary Figure S2.** **Sequence positions in the gene of the five key mutations of ECO_acsA.** (**A**) The sequence of mutation position in ELIM_c1031 (mutation site: -356T, type of mutation: insertion) encoding integrase protein. (**B**) The sequence of mutation position in ELIM_c1038 (mutation site: G133A, type of mutation: SNV). (**C**) The sequence of mutation position in ELIM_c1073 (mutation site: T408G, type of mutation: SNV), *dam.* (**D**) The sequence of mutation positions in ELIM_c1653 (mutation site: C290A, type of mutation: SNV) and _c1654 (mutation site: -216A, type of mutation: insertion), *acsA* and *cooC2*, respectively. The mutation in ELIM_c1653, *acsA*, was identified only in ECO_acsA strain.


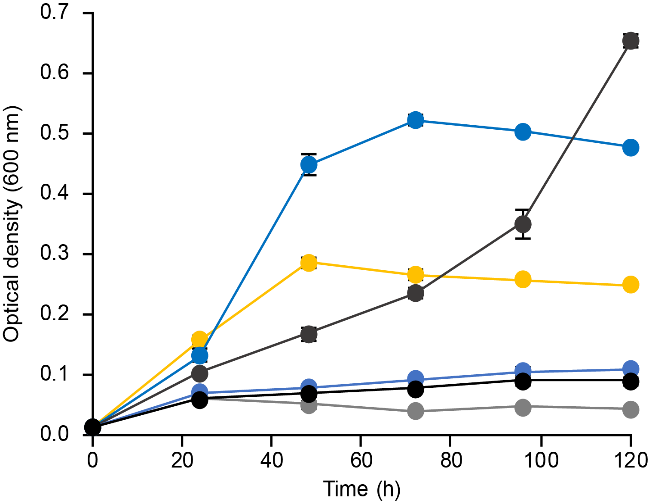


**Supplementary Figure S3.** **The growth profile of ECO_acsA.** The isolated single clone from the adapted strain was cultured under 0% (gray circle), 20% (yellow circle), 40% (light blue circle), 60% (dark gray circle), 80% (blue circle), and 100% (black circle) CO conditions, which are balanced with N_2_, and then the optical density (600 nm) was measured over 120 h. Each circle indicates sampling point for the optical density (600 nm) measurement.


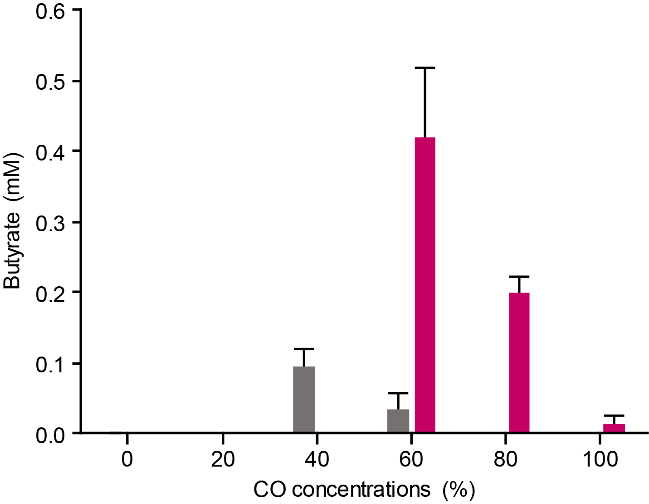


**Supplementary Figure S4.** **The butyrate production of ECO.** The butyrate production of ECO (pink bar) compared to the parental strain (gray bar) under 0%, 20%, 40%, 60%, 80%, and 100% CO conditions, which are balanced with N_2_. Error bars indicate standard deviation of biological triplicates.

**Supplementary Table S1.** **Primers for confirmation of the sequence in key mutation positions of the single clone**

| Primer | Sequence (5’ → 3’) | Note |
| --- | --- | --- |
| c1031_mut_confirm_F | CAAAAGCCCTTAAATAGGCG | For amplification of ELIM_c1031 region containing mutation site (577 bp) and sequencing of the mutation site using only forward primer |
| c1031_mut_confirm_R | AATGTCAAGCTGTATTTGCG |  |
| c1073_mut_confirm_F | GTGTCTGGCAAATGGTATTG | For amplification of ELIM_c1031 region containing mutation site (968 bp) and sequencing of the mutation site using only forward primer |
| c1073_mut_confirm_R | TTTAATCACGGTATCACCCC |  |
| c1038_mut_confirm_F | GTGTGAACATTGCACAGTC | For amplification of ELIM_c1031 region containing mutation site (945 bp) and sequencing of the mutation site using only forward primer |
| c1038_mut_confirm_R | CAATCTCTGGAAAAAGCTGC |  |
| Final_confirm_foracsA_HA_F | ACTGGCACTTGACACCGC | For amplification of ELIM_c1031 region containing mutation site (3,976 bp) |
| Final_confirm_forcooC2_HA_R | ATAACAGCAACACCTGGG |  |
| acsA_mut_confirm_F | ATGCAGACTCCGTTCTGG | For sequencing of mutation site in *acsA* |
| cooc2_mut_confirm_F | GTTAAAGAATGGACTGGC | For sequencing of mutation site im *cooC2* |

**Supplementary Table S2.** **Primers for constructing the acetoin producing strains**

| Primer | Sequence (5’ → 3’) | Note |
| --- | --- | --- |
| alsS_F | CCATACGCGTGGATCCCTCGAGATGTTGACAAAAGCAACAAAAGAACAAAAATC | For amplification of *alsS* and cloning pJIR750_alsD_alsS |
| alsS_R | ATGATTACGAATTCGAGCTCCTAGAGAGCTTTCGTTTTCATGAGTTCC |  |
| alsD_F | CGGTACCCGGGGATCCACGCGTATGGAAACTAATAGCTCGTGCGATTG | For amplification of *alsD* and cloning pJIR750_alsD |
| alsD_R | ATGCCTGCAGGTCGACCTAACCCTCAGCCGCACGGATAG |  |
| alsD_P1121_U1121_F | ACATCTCGAGGGATCCCATTTACCGGGCCAAGC | For cloning pJIR750_alsD_U1121_ P1121_alsS |
| alsD_P_U1121_univ | TAGTTTCCATACGCGTTTCCTCCTTGAAACAAGACGTTCTGAG |  |
| alsS_P2885_U1121_F3 | CCGGTAAATGGGATCCTTTAAGCGTGAAGTGAAAAGAATGG | For cloning pJIR750_alsD_U1121-P1121_P2885_U1121_alsS |
| alsS_P_U1121_univ | TTGTCAACATCTCGAGTTCCTCCTTGAAACAAGACGTTCTGAG |  |
| PU_confirm_F | CAGTTAAACGGCCGACTGCTTG | For confirmation of transformant by PCR (902 bp) |
| PU_confirm_R | GTCCAGCCGGTTAAACGTGC |  |

**Supplementary Table S3. Mutations in the evolved populations and ECO_acsA**

| Samples | Locus tag | Position | Type | Reference | Allele | AA change |
| --- | --- | --- | --- | --- | --- | --- |
| ECO_acsA, 2, 3, 4 | ELIM_c1653 | 1,832,907 | SNV | C | T | Ala^92^Val |
|  |  | 1,832,922 | SNV | C | A | Ala^97^Glu |
| 1, 2, 3, 4 | ELIM_c1031 | 1,126,411 | Insertion | - | T | Asn^119^LysfsX132 |
| 1, 2, 3, 4 | Intergenic | 1,970,647 | SNV | G | A | - |
| 1, 2, 3 | ELIM_c1654 | 1,834,784 | Insertion | - | A | Ala^72^AlafsX92 |
| 1, 2, 3 | ELIM_c3581 | 3,896,831 | SNV | C | A | Asp^66^Tyr |
| 1, 2, 3 | Intergenic | 1,972,135 | SNV | T | C | - |
| 1, 2, 4 | ELIM_c1038 | 1,130,590 | SNV | G | A | Glu^48^Lys |
| 1, 2, 4 | ELIM_c1073 | 1,159,055 | SNV | T | G | Tyr^136^X |
| 1, 4 | ELIM_c0527 | 588,552 | Deletion | C | - | Gly^279^ValfsX282 |
| 1 | ELIM_c0236 | 256,802 | SNV | G | T | Ser^348^X |
| 1 | ELIM_c0337 | 370,333 | SNV | C | G | Glu^315^Gln |
| 1 | ELIM_c0437 | 483,053 | SNV | G | A | Ala^185^Val |
| 1 | ELIM_c0530 | 592,464 | SNV | G | A | Ile^774^Ile |
| 1 | ELIM_c0659 | 726,708 | SNV | G | C | Pro^74^Arg |
|  |  | 726,714 | SNV | T | C | Asp^72^Gly |
| 1 | ELIM_c0672 | 739,966 | SNV | C | A | Ala^88^Ser |
| 1 | ELIM_c0750 | 832,772 | SNV | G | C | Ala^326^Ala |
| 1 | ELIM_c0854 | 938,560 | SNV | A | G | Lys^490^Arg |
| 1 | ELIM_c0866 | 952,049 | SNV | G | A | Val^789^Val |
| 1 | ELIM_c1063 | 1,148,482 | SNV | G | T | Gly^741^Trp |
| 1 | ELIM_c1325 | 1,436,020 | SNV | A | G | Ile^865^Thr |
| 1 | ELIM_c2814 | 3,101,419 | SNV | C | A | Ala^63^Ala |
| 1 | ELIM_c2882 | 3,162,881 | SNV | G | A | Gly^38^Arg |
| 1 | ELIM_c2942 | 3,240,199 | SNV | C | A | Arg^143^Arg |
| 1 | ELIM_c3150 | 3,443,977 | SNV | T | C | Asp^310^Gly |
| 1 | ELIM_c3386 | 3,699,117 | SNV | T | A | Leu^144^X |
| 1 | ELIM_c3427 | 3,747,388 | SNV | G | T | Asp^56^Tyr |
| 1 | ELIM_c3691 | 3,999,914 | SNV | C | A | Met^194^Ile |
| 1 | Intergenic | 3,309,964 | SNV | A | T | - |
| 2 | ELIM_c2071 | 2,255,729 | SNV | C | A | Gly^14^Val |
| 2 | ELIM_c2621 | 2,852,312 | SNV | G | C | Leu^152^Leu |
| 2 | ELIM_c3002 | 3,306,141 | SNV | C | T | Ser^47^Ser |
| 2 | Intergenic | 3,183,238 | SNV | G | C | - |
| 2 | Intergenic | 3,305,753 | SNV | G | A | - |
| 3 | ELIM_c0293 | 322,696 | SNV | G | T | Ile1^70^Ile |
| 4 | ELIM_c0006 | 5,401 | SNV | G | T | His^99^Asn |
| 4 | ELIM_c1330 | 1,446,536 | SNV | G | A | Val^392^Val |
| 4 | Intergenic | 1,946,081 | SNV | G | C | - |

**Supplementary Table S4. Mutations in the ECO_acsA strain**

| Locus tag | Gene | Mutation (Type) | AA change | Description |
| --- | --- | --- | --- | --- |
| ELIM_c0006 | - | G1265T (SNV) | Ala^422^Glu | Gp11 |
| ELIM_c2214 | - | -413G (insertion) | Arg^138^Arg | Hypothetical protein |
| ELIM_c2227 | - | G82T (SNV) | Ala^28^Ser | Terminase |
| ELIM_c1653 | *acsA* | C290A (SNV) | Ala^97^Glu | CODH catalytic subunit |
| Intergenic | - | C2393145- (deletion) | - | - |
|  | - | A2393154- (deletion) | - | - |
